# Supplementary material for: Flagellin Improves the Immune Response of an Infectious Bursal Disease Virus (IBDV) Subunit Vaccine
Source: Vaccines (Basel). 2022 Oct 22;10(11):1780. doi: 10.3390/vaccines10111780 (PMC9695526; doi:10.3390/vaccines10111780)
Supplement: Supplementary file 1 [file vaccines-10-01780-s001.zip › vaccines-1942116-supplementary.pdf]

# Supplementary Material

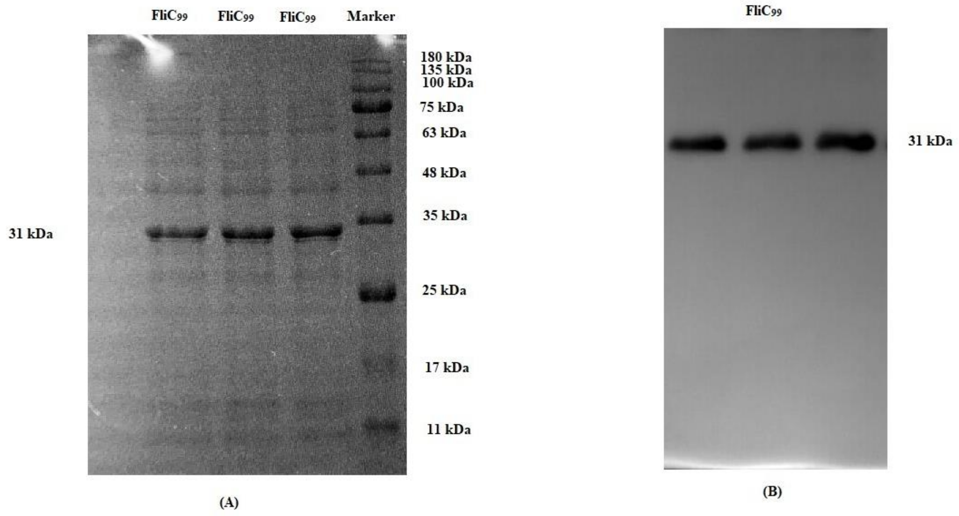


Figure S1: (A) Gel photo of the SDS-PAGE of the recombinant FliC$_{99,}$ (B) Western Blot membrane photo of the recombinant FliC$_{99}$

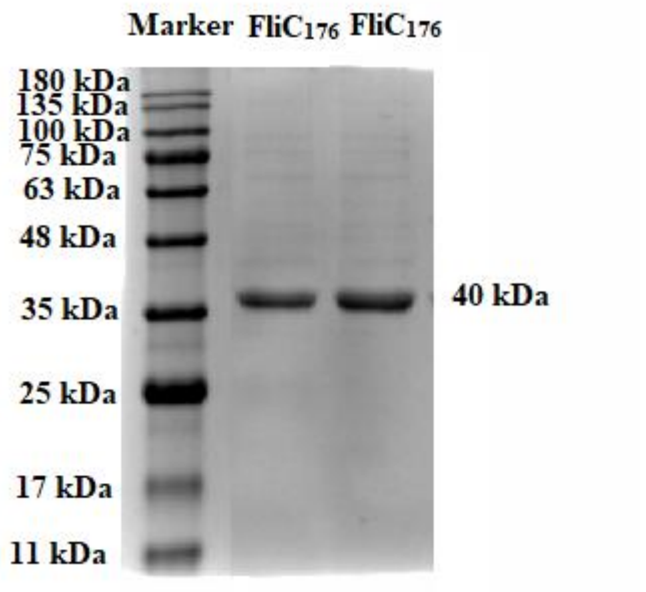


Figure S2: Gel photo of the SDS-PAGE of the recombinant FliC$_{176}$

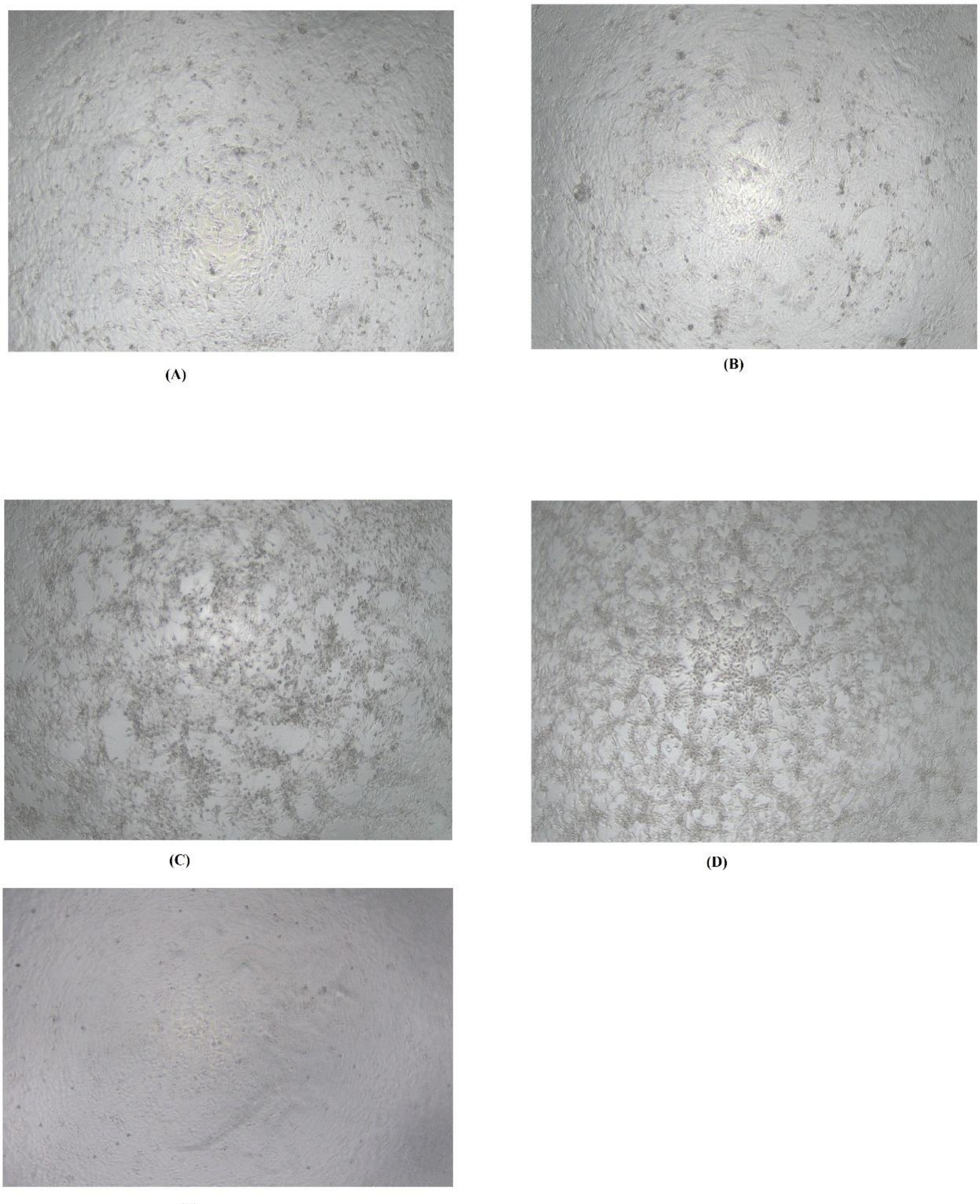


Figure S3: (A) Neutralization by FliC$_{176}$-*t*VP2, (B) Neutralization by FliC$_{99}$-*t*VP2 (C) Virus cytopathic effect (D) Positive Control (E) Negative Control
